# Germline retinoblastoma: estimating risk and counselling the family

**Published:** 2018-06-03

**Authors:** Elisabeth Rosser, Mandeep S Sagoo

**Affiliations:** 1 Consultant Clinical Geneticist: Clinical Genetics Unit, Great Ormond Street Hospital, Great Ormond Street, London, UK.; 2 Retinoblastoma Service: Royal London Hospital; Ocular Oncology Service NIHR Biomedical Research Centre for Ophthalmology, Moorfields Eye Hospital and UCL Institute of Ophthalmology, London UK.

**Even when a diagnosis of germline retinoblastoma cannot be confirmed by genetic testing, the risk to family can still be estimated and used to plan follow-up eye examinations.**

**Figure:**
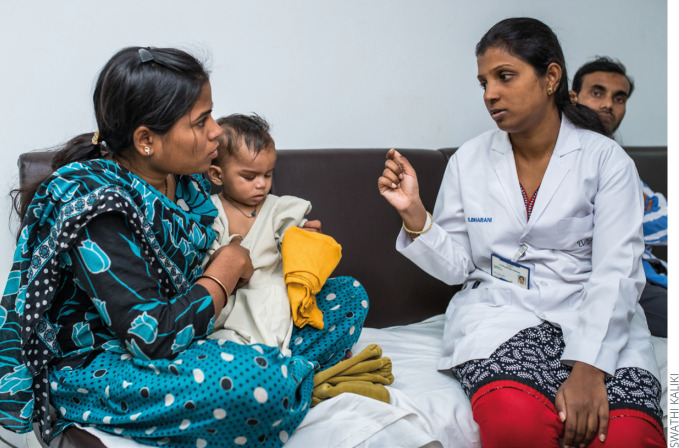
If a child has germline retinoblastoma, siblings are also at risk. INDIA

Genetic laboratories can detect many mutations in the retinoblastoma gene and genetic testing is used to assess whether individuals - and their relatives - have a significant chance of developing retinoblastoma.

However, genetic testing is not available in many treatment centres in low- and middle-income countries. In this situation, clinicians should evaluate the likelihood that a child has germline retinoblastoma, based on the clinical presentation and family history:

- Germline retinoblastoma occurs at a younger age (median of 15 months)
- Tumours are usually bilateral and multifocal, but may be unilateral
- Others in the family are affected, either with retinoblastoma, or, rarely, with a second tumour
- There is a history of childhood enucleation in a parent or other close relative
- Parents or siblings may have a retinoma on examination (Figure 1, p. 7).

If a child has **germline** retinoblastoma (see p. 7), his or her siblings (and potential offspring) are at risk of developing the condition. They must undergo a full retinal examination at regular intervals so that any tumours can be detected and treated as early as possible.

If a child presents with unilateral retinoblastoma, always examine the second eye very carefully until the child is 5 years of age so any signs of bilateral retinoblastoma can be identified and treated.

**Note:** If a child presents with unilateral retinoblastoma, always examine the second eye very carefully so any signs of bilateral retinoblastoma can be identified and treated early

## Estimating the risk of germline disease

**Figure 1:**
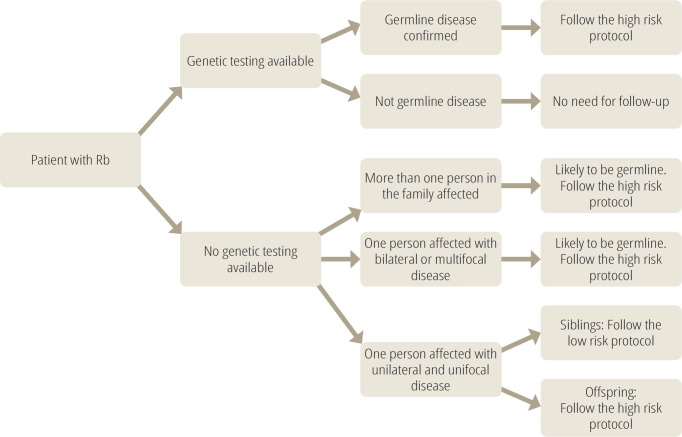
Flow chart. Assess the risk to family members using Table 1 then follow the suggested screening protocol given in Table 2

To support decision making in the absence of genetic testing, an algorithm (Figure 1) can be used to estimate the risk to family members if a child has retinoblastoma. The algorithm was developed by reviewing the outcomes of children in our unit who had undergone genetic testing.

Table 1 shows the estimated risk that a sibling, child or more distant relative of someone with retinoblastoma will also develop a tumour in the retina. Important factors are whether more than one person in the family has retinoblastoma and, if there is no-one else affected on the family, whether the affected person has multifocal/bilateral or unilateral retinoblastoma The categories are grouped by the level of risk, with more than 1% being considered ‘high risk’ and 1% or less ‘low risk’.

Use Table 1 to decide whether the relationship to the affected person and the clinical scenario merits the use of a ‘high-risk’ (orange) or ‘low-risk’ (green) screening protocol. Table 2 gives the suggested screening protocols, which set out when, and how often, family members must undergo detailed retinal examinations to look for early signs of a tumour.

It is important to speak to parents or carers and explain the risk to their child and the fact that siblings and the child's future children may also develop retinoblastoma. Emphasise that the regular eye examinations suggested in Table 2 will help to diagnose any disease early so that it can be treated successfully. Listen to parents concerns. Ensure they know when and where to bring family members for examinations and allow enough time to answer any questions they may have.

## Second tumours

People with germline retinoblastoma are also at risk of developing other tumours later in life. These second tumours are most commonly osteosarcomas, soft tissue sarcomas or melanomas. The risk is increased if the child was treated with external beam radiotherapy. These second tumours most commonly occur between 10 and 50 years of age and can occur anywhere in the body. There is no effective screening for second tumours, so it is important for the patient and their medical team to be aware that they may occur. Sun protection is important, as is regular checking of the skin for melanomas.

**Table 1:** Estimated risks of developing retinoblastoma in siblings and offspring of a child presenting with retinoblastoma

	More than one affected person in family	The child has bilateral or multifocal retinoblastoma	The child has unilateral unifocal retinoblastoma
**Siblings** of Rb child	**50%**	**5%**	**1%**
**Offspring** of child who had Rb	**50%**	**50%**	**5–10%**
**More distant relatives** of child with Rb	Need to assess the family tree, may be at risk	Not at increased risk	Not at risk
□ High risk □ Low risk			

**Table 2:** High risk and low risk screening protocols for offspring or siblings of children with retinoblastoma

Age of sibling or offspring	High risk	Low risk
2 weeks	Screen by 2 weeks	
4 weeks		Screen by 4 weeks
Up to 6 months	4 weekly	6 weekly
6–12 months	Every 4–6 weeks	9 months and 12 months
1–2 years	Every 2 months until 18 months Then at 21 months and 24 months	16 and 22 months
2–3 years	Every 4 months	Every 6 months
After 3 years	Stop, unless there is a family history of late onset	Stop

